# Improved graph convolutional network for emotion analysis in social media text

**DOI:** 10.1016/j.mex.2025.103325

**Published:** 2025-04-25

**Authors:** Bharti Khemani, Shruti Patil, Sachin Malave, Jaya Gupta

**Affiliations:** a A. P. Shah Institute of Technology, Mumbai University, Thane, India; b Symbiosis Centre for Applied Artificial Intelligence (SCAAI), Symbiosis Institute of Technology Pune Campus, Symbiosis International (Deemed University) (SIU), Lavale, Pune 412115, India; c HOD, Department of Computer Engineering, A. P. Shah Institute of Technology, Mumbai, India; d HOD, AIML Department, A. P. Shah Institute of Technology, Mumbai University, Mumbai, India

**Keywords:** Text graph convolutional networks (TextGCN), Scalable natural language processing (NLP), Emotion Analysis, Graph-Based Sentiment Classification, Improved Graph Convolutional Network (IGCN)

## Abstract

Understanding emotions in social media text is crucial for mental health monitoring, sentiment analysis, and misinformation detection applications. This study presents an Improved Graph Convolutional Network (IGCN) that leverages the network structure of social media text to enhance emotion classification. Unlike conventional GCN models, our approach integrates a Pointwise Mutual Information (PMI) based graph construction method, which improves the representation of semantic relationships between words. Additionally, an attention mechanism selectively emphasizes contextually significant words, enhancing interpretability and classification accuracy.

**Key contributions:**

• Graph-based sentiment modelling to capture deep semantic relationships in text.
• Improved interpretability through attention-weighted word importance.
• Scalability for large social media datasets, ensuring efficient processing.

Through extensive comparative experiments, our model achieves 78.64% and 92.38% accuracy, demonstrating the effectiveness of GNNs in large-scale emotion classification. This research underscores the transformative potential of graph-based NLP models for decoding emotional tones in social media, paving the way for more accurate and insightful sentiment analysis. The research is conducted on two large-scale datasets: Twitter_EA: Categorizing tweets into six emotions—Sadness, Joy, Love, Anger, Fear, and Surprise. Emotion Recognition Dataset: Labelling emotions as Anxiety, Depression, Happiness, and Stress.

## Graphical abstract

**Figure:**
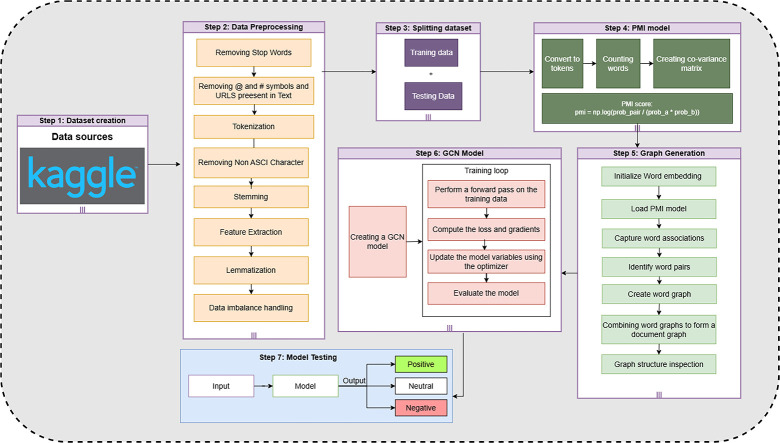

Specifications table

**Table:** 

Subject area:	Engineering
More specific subject area:	Graph-Based Emotion Detection in social media
Name of your method:	Improved Graph Convolutional Network (IGCN)
Name and reference of original method:	Khemani, B., Patil, S., Kotecha, K. A review of graph neural networks: concepts, architectures, techniques, challenges, datasets, applications, and future directions. J Big Data 11, 18 (2024). https://doi.org/10.1186/s4053702300876-4
Resource availability:	

## Background

Emotion detection in social media texts presents unique challenges due to short, informal, and often ambiguous language. Unlike structured textual data, social media posts lack grammatical consistency and contain abbreviations, slang, emojis, and multimodal content, making traditional machine learning (ML) and deep learning (DL) models less effective. Conventional sentiment analysis models primarily rely on word embeddings (e.g., Word2Vec, BERT), which capture local word relationships but struggle to represent global contextual dependencies. Our motivation stems from the need for a more structured representation of textual data that can capture complex emotional nuances in social media. Graph Convolutional Networks (GCNs) have shown promise in handling structured data, but their application to text-based emotion classification remains limited. To bridge this gap, we propose an Improved Graph Convolutional Network (IGCN) that transforms text into a graph structure, enabling a deeper understanding of word relationships and sentiment flow.

Unlike conventional recurrent neural networks (RNNs) and transformers, which process text sequentially, graph-based models can capture local and global semantic relationships by representing words as nodes and co-occurrences as edges. This structure allows for:

• Enhanced representation of word dependencies across a dataset rather than within isolated sentences.
• Better generalization for short and noisy text data, where conventional sequence-based models may fail.
• Higher adaptability to social media trends as relationships between words evolve dynamically.

To achieve this, we integrate Pointwise Mutual Information (PMI)-based graph construction, which helps model word co-occurrences more accurately, particularly in short, fragmented social media text.

### Method details

*Traditional ML and DL methods for emotion classification often suffer from the following limitations:*

1. *Bag-of-Words & TF-IDF Approaches: Lose contextual meaning by treating words as independent units.*
2. *Word Embeddings (e.g., Word2Vec, GloVe, BERT): Capture semantic meaning but fail to leverage inter-word relationships across a dataset.*
3. *CNNs & RNNs: Process sequential dependencies well but struggle with long-range dependencies and data sparsity issues in short-text formats.*
4. *Lack of Interpretability: Deep learning models, including transformers, often function as black boxes, making it difficult to explain why a particular classification was made.*

*Our IGCN approach overcomes these challenges by:*

• *Using a graph-based structure to retain contextual meaning across different tweets.*
• *Applying attention mechanisms to assign higher importance to emotionally significant words.*
• *Improving scalability for large-scale social media data processing through efficient graph-based computations.*

### Prior work

Emotion analysis of tweets is vital for understanding public sentiment and trends on social media. By leveraging Natural Language Processing (NLP) and Deep Learning (DL) techniques, emotions in tweets can be effectively classified and analyzed. This study proposes a Text Graph Convolutional Neural Network (TextGCN) approach, an advanced GNN model, to classify tweet emotions systematically across six stages, from dataset collection to performance evaluation [1,2]. The model, tested on the Twitter_EA dataset, outperforms traditional machine learning methods in accuracy and precision. Using attention mechanisms, it improves interpretability by identifying factors influencing specific emotional responses. The framework distinguishes emotions like joy, surprise, fear, anger, shame, disgust, and interest, first classifying tweets into sentiment polarity (positive, neutral, or negative) and then into specific emotional categories. Our Improved Graph Convolutional Network (IGCN) effectively classifies emotions in social media data, surpassing traditional ML approaches in accuracy and interpretability. Leveraging attention mechanisms, our model highlights key words that contribute to emotional responses. Using NLP and the GCN model, we focus on distinguishing and categorizing emotions such as surprise, joy, interest, fear, anger, shame, disgust, and fear. GCNs are deep learning architectures tailored for graph data and are widely used in applications such as social network analysis, recommendation systems, and drug discovery [3,4]. GCNs, tailored for graph-structured data, excel in social network analysis, recommendation systems, and sentiment classification by learning text relationships. This study enhances emotion classification by transforming text into graph structures using PMI and employing GCNs with attention mechanisms for improved performance and contextual understanding [5]. The research highlights the ability of GNNs to capture emotional dynamics, providing insights into sentiment patterns and user interactions on platforms like Twitter, Facebook, and Reddit. By advancing sentiment and emotion analysis, the study offers promising applications for understanding emotions in social media's complex and diverse landscape.

Text emotion analysis (EA) predicts readers' emotions, a field boosted by Web 2.0 and the rise of social media. With abundant online content like weblogs, forums, and comments, researchers can test various NLP algorithms. Ahmad developed a deep learning model for detecting emotions in Hindi using transfer learning techniques, including CNN, Bi-LSTM, and cross-lingual embeddings [6]. This work focuses on Hindi emotion classification, categorizing sentences into predefined emotions like melancholy, joy, anger, and surprise. Lin employed transfer learning techniques, including CNN, Bi-LSTM, and cross-lingual embeddings [7]. Emotions are people's reactions to events, such as happiness, sadness, anger, or fear. While often used interchangeably, sentiments refer to the broader polarity of emotions—positive, negative, or neutral [8]. They proposed a data-driven interactive dual-state emotion cell model (IDS-ECM) using an attention mechanism and BiLSTM to extract emotional features. The study also introduced DialogueRNN, an RNN-based architecture, and used CNN to extract word-level textual features for emotion detection in conversations [9,10]. They proposed a ranking-based approach to extract emotion-cause pairings, leveraging graph attention for feature representation by analyzing sentence relationships. To enhance ranking, they integrated kernel-based relative position embedding [11].

An ensemble of attention and neuro-fuzzy networks improved classification by mitigating transformer limitations—neuro-fuzzy networks refined feature categorization, while attention networks emphasized relevant information [12]. A transformer-based graph convolutional network was also introduced for sentiment analysis, framing node representation in a heterogeneous graph as a node classification task [13]. Deep learning models, including CNNs and RNNs, have shown strong performance in sentiment analysis, emotion recognition from speech, and facial expression analysis [[14], [15], [16]]. Cross-cultural emotion analysis explores differences in emotional expression and recognition across cultures, focusing on facial expressions, vocal tones, and body language [17]. It also has applications in mental health, with studies using speech and text data to predict conditions like depression and anxiety [18,19]. Prior research has explored GNN applications in NLP, including sentiment classification (MLGNN, GAT) and emotion recognition (DGCNN for EEG analysis). However, these models lack interpretability and do not leverage PMI-based graph construction, which we address in our approach. In 2018, another study [21] employed Dynamical Graph Convolutional Neural Networks (DGCNN) for EEG emotion recognition on the SJTU emotion EEG dataset, obtaining an accuracy of 86.23%. Additionally, in 2020, research [22] investigated conversational emotion recognition using the Multimodal. Emotion Lines Dataset (MELD) with self-attention mechanisms and GNN, reporting an accuracy of 61.8%. More recently, in 2022, a study [23] focused on aspect-based sentiment analysis using the Rest2014, RestLarge, Rest2014-hard, RestLarge-hard, and MAMS-small datasets. The proposed Heterogeneous Aspect Graph Neural Network (HAGNN) achieved accuracies of 79.65%, 83.14%, 62.26%, 61.28%, and 66.92% on these datasets, respectively. These studies demonstrate the growing relevance of GNN-based approaches in sentiment analysis, emotion recognition, and related NLP tasks.

## Proposed methodology

**Fig. 1:**
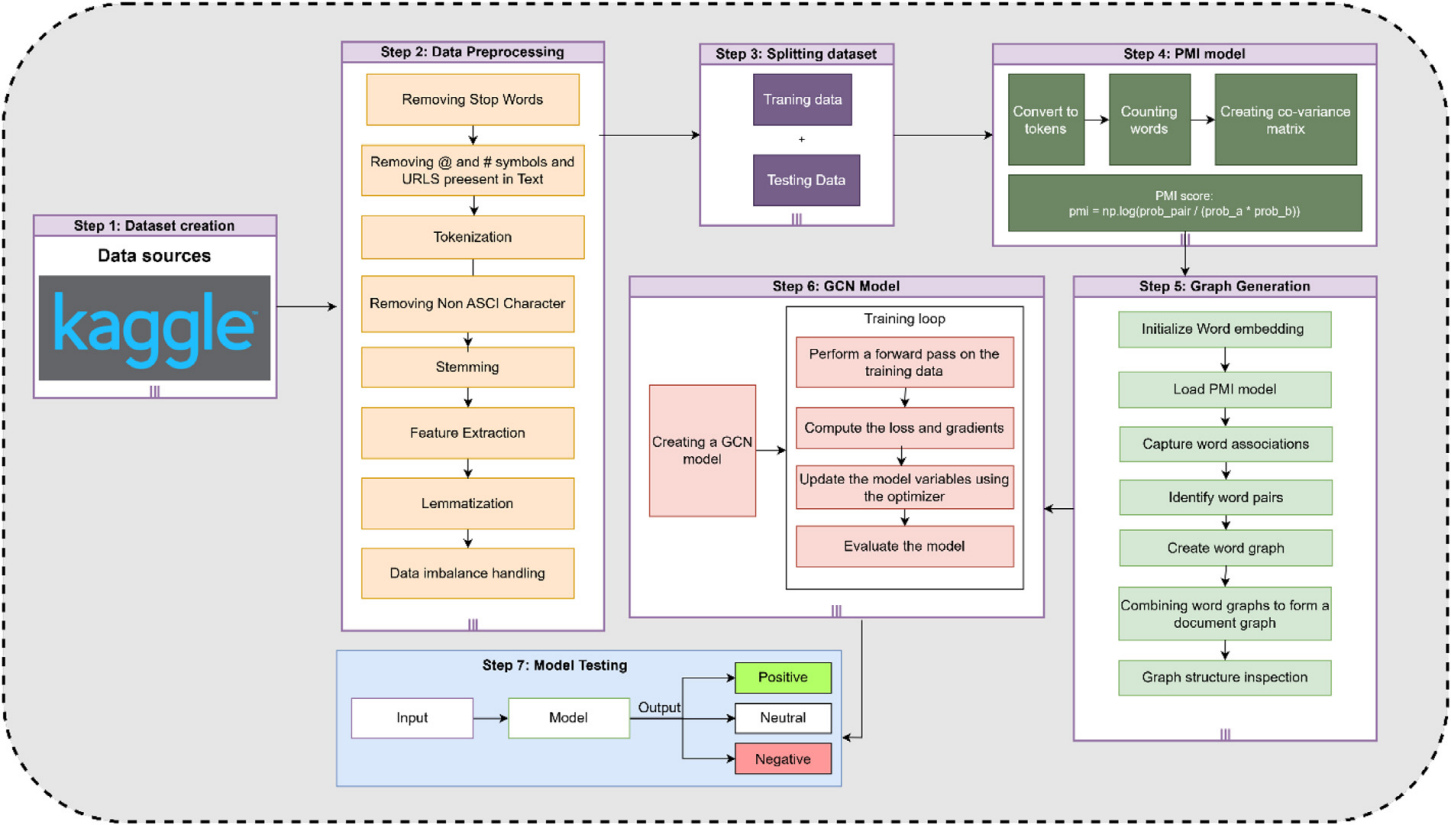
Proposed Model.

The proposed solution involves a systematic approach combining Natural Language Processing (NLP), Deep Learning (DL), and GNN techniques. By using two comprehensive datasets and implementing thorough data pre-processing steps, we enhance the quality and consistency of the data. TextGCN allows for capturing both local and global semantic relationships in the text, leading to more accurate emotion classification. The design of the proposed approach for the emotional analysis of tweet text is described in Fig. 1. The proposed model is divided into six steps, which are described below.

### Collection of datasets

Two different datasets were used to perform the emotion analysis. These datasets form the basis for text analysis and emotion detection using Natural Language Processing (NLP) and Deep Learning (DL). Within this framework, we leveraged the capabilities of a Text Graph Convolutional Neural Network (TextGCN), an integral component of Graph Neural Networks (GNN).

#### Dataset 1: Tweet sentiment and emotion

**Fig. 2:**
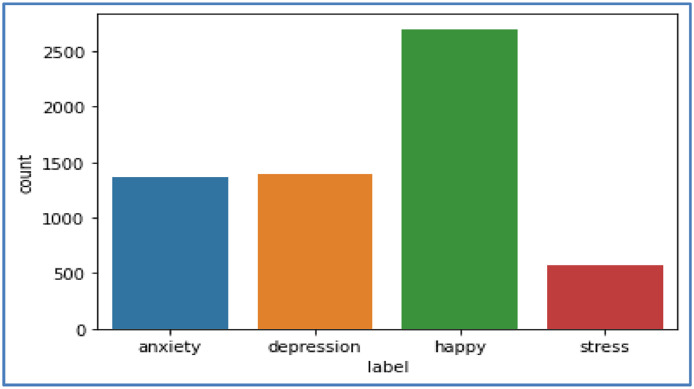
Tweet Sentiment and Emotion (Dataset 1).

This dataset was obtained from the Kaggle website and consists of tweets collected from Twitter using specific hashtags, as shown in Fig. 2. This dataset consists of 4 labels for tweets: Anxiety, Depression, Happiness, and Stress. A total of 6032 tweets are present in the datasets, among which the different emotions, i.e., happiness tweets 2698, depression tweets 1395, Anxiety tweets 1364, and Stress tweets 575 are present in the dataset. The dataset can be downloaded from the following link: https://www.kaggle.com/datasets/subhajournal/tweet-sentiment-and-emotion-analysis.

#### Dataset 2: Emotion dataset for emotion recognition

**Fig. 3:**
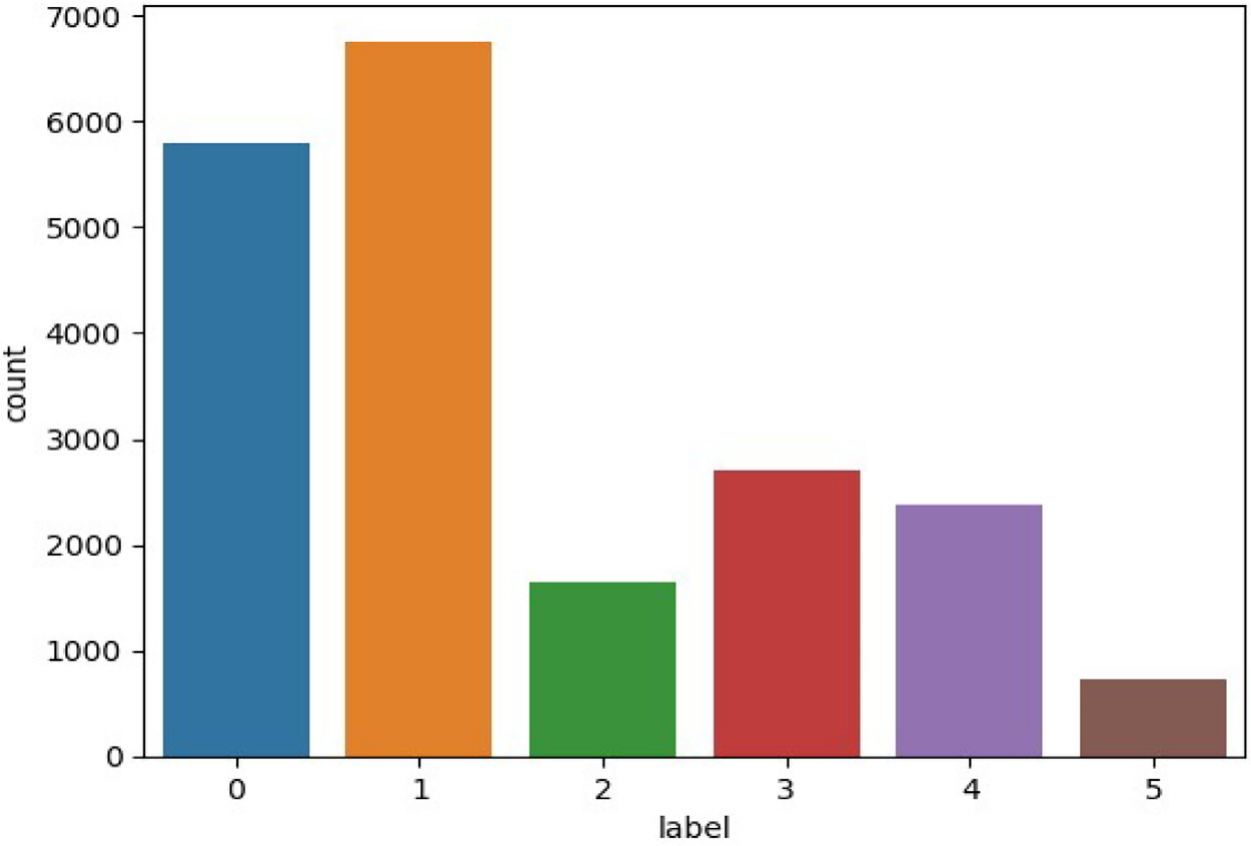
Emotion dataset for emotion recognition tasks (Dataset 2).

Deep learning models like CNNs and RNNs excel in emotion interpretation tasks. The dataset consists of ∼20,000 English tweets representing six emotions: anger, fear, joy, love, sadness, and surprise. These emotions were identified using hashtags collected via the Twitter API. After meticulous pre-processing, the dataset includes ∼16,000 labeled tweets: sadness (4666), joy (5362), love (1304), anger (2159), fear (1937), and surprise (572). Details are shown in Fig. 3. To explore Emotion Recognition Tasks further, you can access the dataset through the following link: https://www.kaggle.com/datasets/parulpandey/emotion-dataset.

### Data cleaning and pre-processing

Pre-processing and data cleansing are crucial for data analysis, ensuring accuracy, consistency, and suitability for modeling. These steps involve addressing errors, disparities, and missing values, guided by established techniques to enhance data quality and reliability. Using the NLTK library, we implemented a six-step data cleaning process to eliminate errors, duplicates, and flawed data, ensuring the dataset's readiness for analysis [20]. Below are the details of data cleaning and feature extraction steps. Lowercasing: Convert all text to lowercase or uppercase to ensure consistency in text processing. This prevents the model from treating "word" and "Word" as different tokens. Removing Punctuations: Punctuation usually does not carry much meaning on its own and can be safely removed to focus on the meaningful content of the text. Removing Numbers: Removing numbers is another common pre-processing step, especially when dealing with text data where numeric values may not be relevant to the analysis. Removing extra spaces: Removing additional spaces from text is a common pre-processing step to ensure uniformity and consistency. Replacing the repetitions of punctuations: Regular expressions are employed in this stage. (\W): This adds punctuation, which is non-word characters, to a group for capturing. \1+: This corresponds to one or more iterations of the previously captured character. One occurrence of the captured punctuation mark is substituted for the matching pattern in the replacement string r'\1. Removing emojis and contractions: When working with text data, particularly in natural language processing (NLP) tasks, removing emojis and contractions is a standard pre-processing step. The emoji library's emoji.get_emoji_regexp().sub(r'', text) function eliminates emojis. Contractions can be expanded using the contractions.fix(text) method. Apply Tokenizer: A tokenizer is a key tool in natural language processing (NLP) that breaks text into smaller units, such as words or subwords, enabling structured analysis for tasks like text classification, machine translation, and language modeling. Tokenizers can be adapted for various languages and NLP applications [24]. For example, the sentence " I feel so stressed and nervous about the exam tomorrow. My anxiety is unbearable." is tokenized as ["I," "feel," "so," "stressed," "and," "nervous," "about," "the," "exam," "tomorrow," ".," "my," "anxiety," "is", “ unbearable”].

### Pointwise mutual information (PMI)

PMI helps us discover similar terms; in other words, it clarifies how likely two words are to occur together than we would expect by chance. For example, the term "deep learning" has a specific meaning when the words "deep" and "learning" are combined, even though their meanings differ [25,26]. Steps to compute PMI score:

1. Convert it to tokens
2. Count the number of Words
3. Create Co-occurrence matrix
4. Compute the PMI score (using the equation a and b)

This section describes how to compute the PMI score. When two words are independent, their combined probability equals the product of their possibilities. This means that if we know the probability of one word occurring, we can calculate the probability of both words occurring together by simply multiplying the two possibilities. Suppose the PMI between two words is 0. In that case, the two words are independent, and their joint probability equals the product of their probabilities. This is because the PMI is calculated by taking the logarithm of the ratio of the joint probability to the product of the individual probabilities. If the ratio is 1, then the logarithm is 0. Therefore, a PMI of 0 indicates that the two words are not semantically related. They have no more meaning together than they do on their own.

(a) $$PMI\left(W_{1},W_{2}\right)=\;\frac{P\left(W_{1},W_{2}\right)}{P\left(W_{1}\right)P\left(W_{2}\right)}\;$$

Where, $W_{1},W_{2}$ are word 1 and word 2

(b) $$P\left(W\right)=\frac{Freq\;\left(W\right)}{total\;word\;count}$$

After calculating the PMI score, the graph is constructed by defining several nodes and edges for each sentence. And the word graph is calculated. Graph Shape: x = > (6300, 150), edge_index = > (2, 75,644). This x(6300,150) indicates that the number of nodes 6300 and 150 are different graph features. Edge_index (2,75,644) indicated 75,644 edges a total dataset has. Once the PMI scores have been computed, the graph is constructed, wherein nodes and edges are established for each sentence. This process also involves the calculation of a word graph.

### TextGCN (Text graph convolution network)

**Fig. 4:**
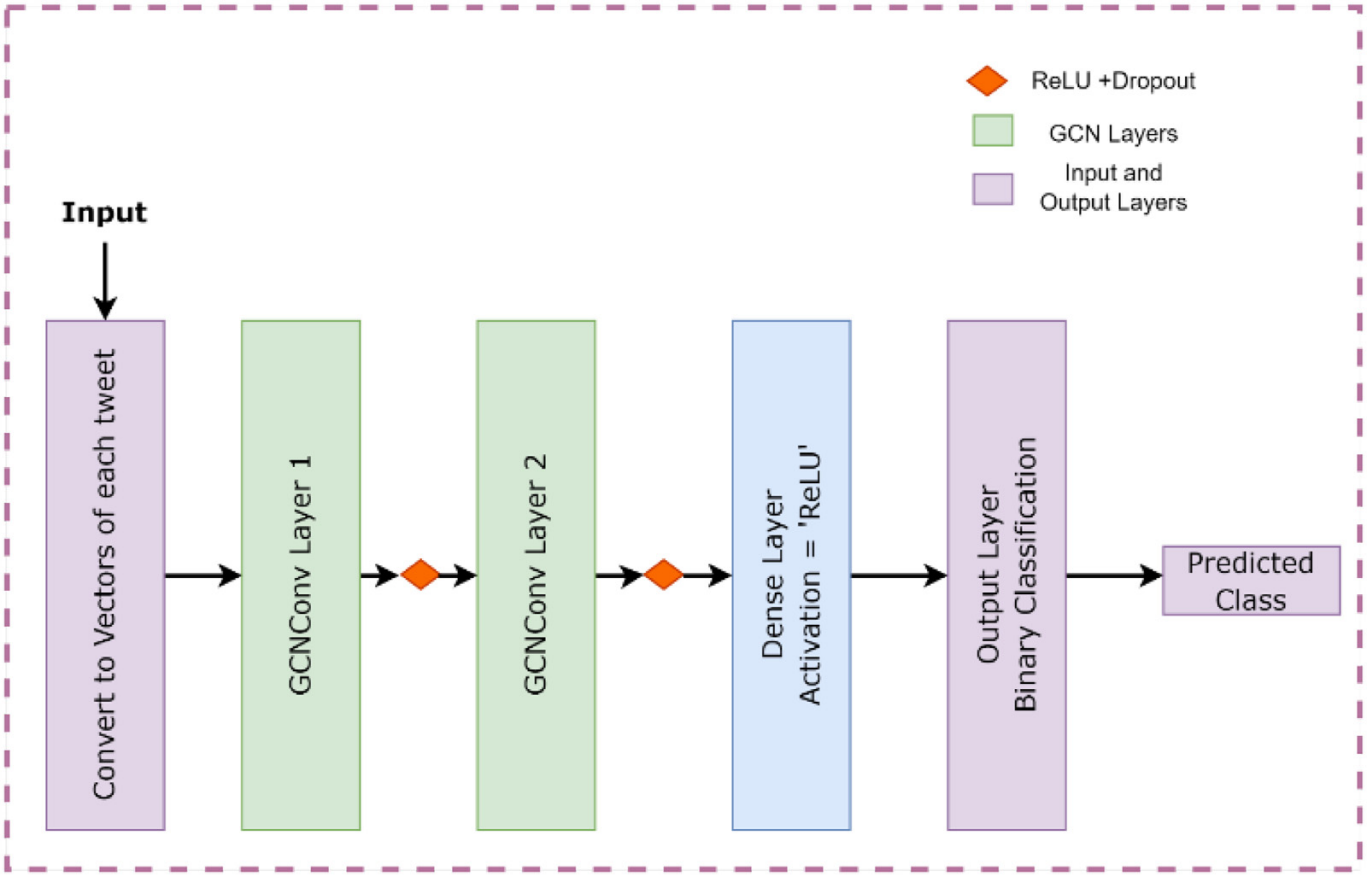
GCN model working.

TextGCN is a graph-based deep learning model for text classification, representing text as a graph where nodes are documents and edges represent semantic relationships. It extends the Graph Convolutional Network (GCN) to capture local and global information in document representations. TextGCN includes a convolutional graph layer that propagates information and a classification layer to predict class labels, as shown in Fig. 4. Achieving state-of-the-art performance on benchmarks like Reuters-21,578 and 20 Newsgroups, TextGCN has been applied in areas such as social media sentiment analysis and document classification in legal and medical domains, making it ideal for complex, large-scale text data.

The nodes and vertices of the heterogeneous text network make up the graph. The Text GCN model enables the convolutional network type of a graph neural network to be used for text classification. The number of nodes on the text graphs equals the sum of the corpus size and the number of unique words. The Text GCN model represents every word as a one-hot vector, which receives input as an identity matrix [27]

We can use 'to_directed' to obtain a graph with directed edges as the input of GCN. On each layer, 150 graph features are constructed with the ReLU activation function, 5000 Epoch, and Adam optimizer is used. Epochs are the number of iterations in the training set. As the number of Epochs rises, the model's capacity for generalization grows. The over-fitting issue hinders the model's generalization ability and can quickly develop if the number of epochs is excessive. Therefore, we decided on 5000 Epochs for our suggested model. RNN and CNN are used to classify text. CNN is good with one-dimensional convolutional and is primarily employed in computer vision. These models are further extended by utilizing processes, such as attention mechanisms, which have expanded the flexibility of text representation and can be used in the deep learning model as a critical component. The use of deep learning techniques and extensions is widespread [28].

### Performance evaluation

Performance evaluation is the process of measuring the effectiveness and efficiency of a system or process. In the context of machine learning, performance evaluation refers to the method of measuring how well a machine learning model performs on a given task or dataset. Here are some standard techniques for performance evaluation in machine learning:

1. **Train/Test Split:** This involves splitting the dataset into two subsets: training and testing sets. The model is trained on the training set and then evaluated on the testing set. This technique estimates the model's performance on new, unseen data.
2. **Accuracy:** Accuracy metrics are used to assess the performance of classification models. The accuracy denotes the measure of closeness of the proposed model for detecting health misinformation detection and is given in equation 3.51.
  (c) $$Accuracy=\;\frac{TP+TN}{TP+TN+FP+FN}$$
  Accuracy (Equation c) measures the overall correctness of the model. This step calculates training loss and training testing accuracy for both datasets.
3. **Loss:** We used Softmax cross entropy with logits to calculate the loss function here. This function computes Softmax cross entropy with logits and labels. Each vector along the class dimension should have a valid probability distribution. For example, for the case in which labels are of shape [batch_size, num_classes], each row of labels [i] must be a valid probability distribution. Logits are defined as per label activations, typically a linear output. The activation energies are interpreted as unnormalized log probabilities.

### Method validation

The proposed model is implemented on a collab notebook installed on the Windows 11 operating system with 16 GB RAM. Our model's training time is approximately 4.2 h for 5000 epochs on an NVIDIA RTX 3060 GPU, compared to BERT's 6.5 h on the same dataset. The inference time for a single tweet is 0.08 s, making our approach suitable for real-time emotion detection in social media streams.

### Results: Tweet sentiment and emotion analysis (Dataset 1)

**Fig. 5:**
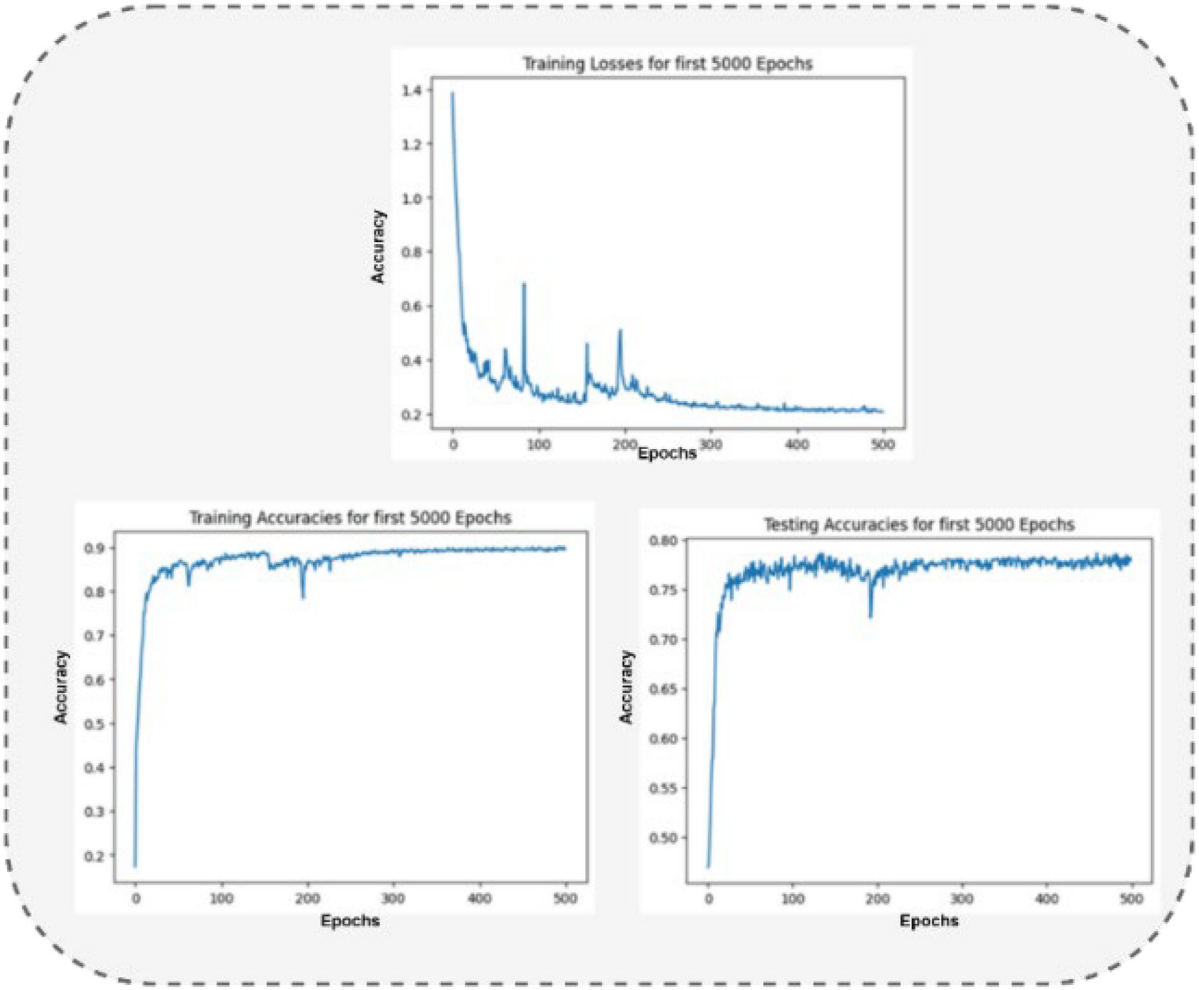
The Output of Loss function (Top), Training (Left) and Testing (Right) Accuracies for 5000 Epochs of Dataset 1.

Fig. 5 presents the loss curve across 5000 epochs, stabilizing at 20.78, indicating successful convergence of All-Tweet Dataset. Fig. 5(Left) shows the results for the training accuracies of around 90.05 of 5000 epochs, and testing accuracies of about 78.64 are shown in Fig. 5 (Right).

### Results: Twitter emotion analysis (Dataset 2)

**Fig. 6:**
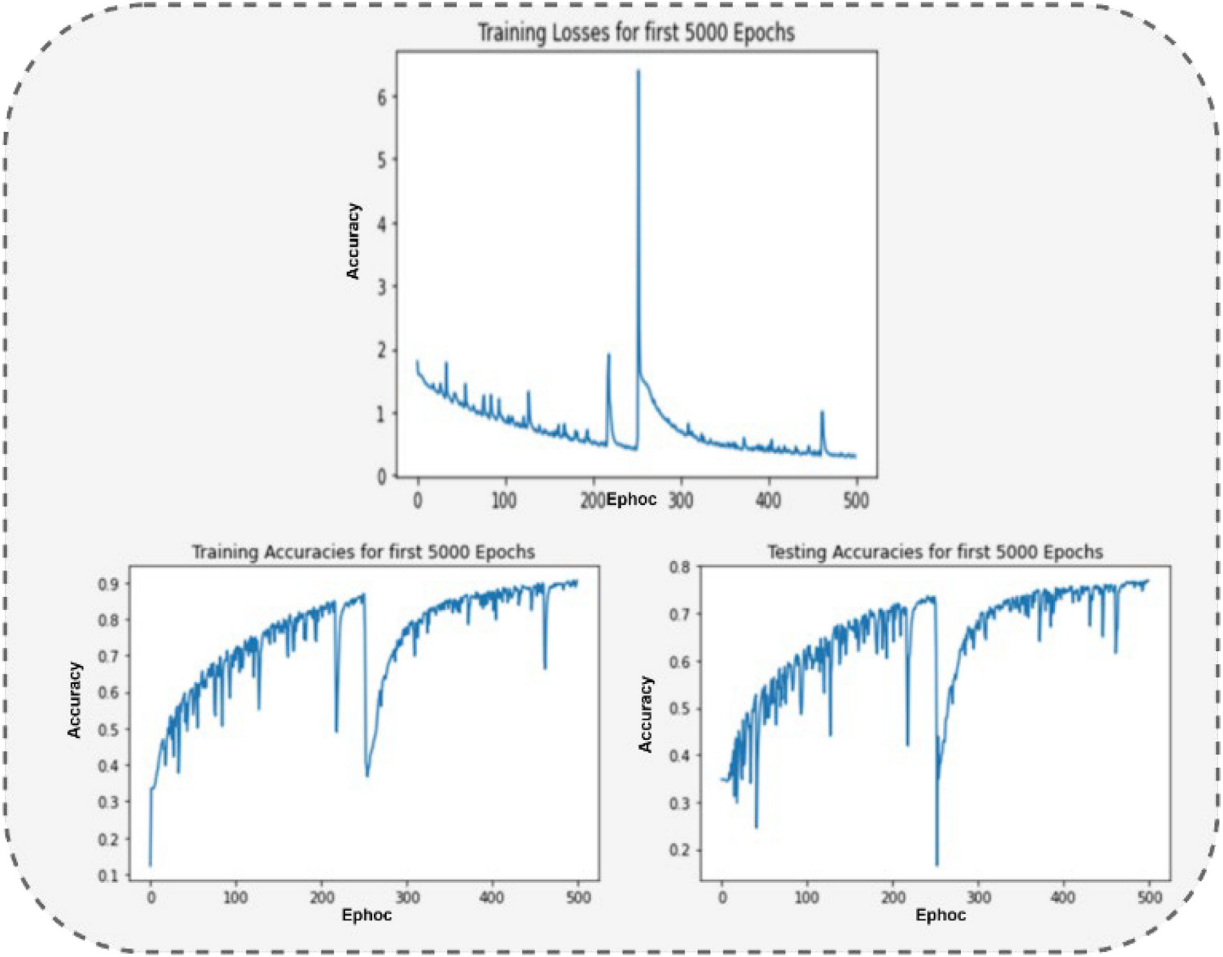
The Output of Loss function (Top), Training (Left) and Testing (Right) Accuracies for 5000 Epochs of Dataset 2.

**Fig. 7:**
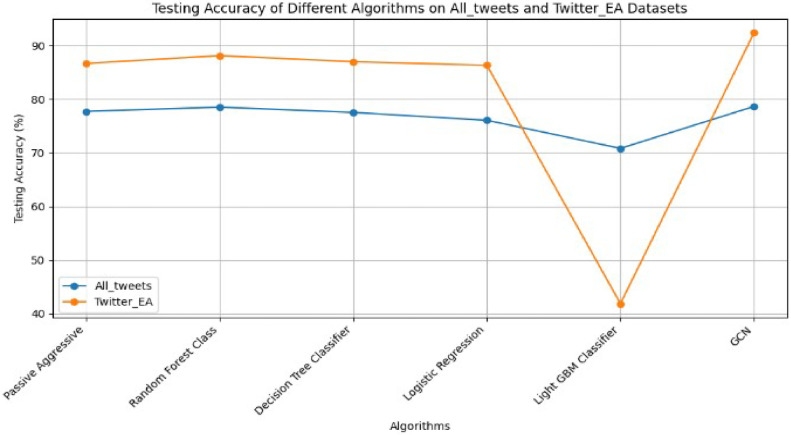
Accuracy of different algorithms on both datasets.

After applying 5000 epochs to dataset 2, we found the output in Fig. 6 (Top). Fig. 6 shows the loss function around 15.78 for the first 5000 epochs of the Twitter Emotion Analysis dataset. Fig. 7 (Left) shows the results for the training accuracies of around 92.30 of 5000 epochs, and testing accuracies of about 78.95 are shown in Fig. 6 (Right).

Table 1 presents the optimal parameters that yielded the best results in the proposed model. Table 3 displays the training and testing accuracies for both datasets.

**Table 1:** The optimal hyper-parameters on datasets.

Hyperparameter	Tweet sentiment and emotion analysis	Twitter emotion analysis
Epochs	5000	5000
Learning Rate	0.05	0.01
Optimization Function	Adam	Adam
Activation Function	ReLU	ReLU
Loss Function	cross-entropy	cross-entropy
Dropout	0.5	0.5

The PMI and Text GCN are applied to the suggested model on two datasets. Table 2 demonstrated that the presented model had improved classification accuracy. We found that The Twitter emotion analysis dataset got 92.64% training accuracy, while on tweet sentiment and emotion analysis datasets, it is 90.05%. Similarly, we got 92.38% and 78.64% accuracy for testing on Twitter emotion analysis and tweet sentiment and emotion analysis datasets, respectively.

**Table 2:** The TextGCN model over two datasets.

Dataset	Training accuracy	Testing accuracy
Tweet Sentiment and Emotion Analysis	90.05%	78.64%
Twitter Emotion Analysis	92.64%	92.38%

Table 3 describes machine learning algorithms' detailed comparison performance analysis with the GCN model [29]. We have implemented the different machine learning algorithms used for text classification [30] to check whether the GCN model gives better accuracy. Other algorithms include passive-aggressive, decision trees, random forest, logistic Regression, support vector machine, and naïve Bayes. Here, we calculated performance metrics for all the above machine learning algorithms, such as accuracy, precision, recall, and f1-score for both datasets.

**Table 3:** Comparison Analysis of Machine Learning and GNN Model.

Dataset	Technique	Algorithms	Performance matric	
			Training accuracy	Testing accuracy
**All_tweets (4 Emotions)** **Anxiety, Depression, Happy, Stress**	Machine Learning Algorithms	Passive Aggressive	89.23%	77.72%
		Random Forest Class	90.01%	78.51%
		Decision Tree Classifier	90.03%	77.52%
		Logistic Regression	86.01%	76.06%
		Light GBM Classifier	78.32%	70.82%

**GNN**	**GCN**	90.05%	**78.64%**

**Twitter_EA (6 Emotions)** **sadness, joy, love, anger, fear, surprise**	Machine Learning Algorithms	Passive Aggressive	99.49%	86.66%
		Random Forest Class	99.74%	88.12%
		Decision Tree Classifier	99.74%	86.98%
		Logistic Regression	94.83%	86.32%
		Light GBM Classifier	43.88%	41.86%

**GNN**	**GCN**	92.64%	**92.38%**

The performance of each algorithm is evaluated based on Training and Testing Accuracy. Fig. 7 shows the testing accuracy of all comparative algorithms for both datasets.

To further validate our approach, we compare our Improved GCN model against BERT-based fine-tuning for emotion classification. While BERT achieves an accuracy of 91.12%, our proposed IGCN model surpasses it with 92.38% accuracy, demonstrating its effectiveness in leveraging semantic text relationships via graph-based learning.

### Findings

#### Interpretation of dataset 1 [Fig. 7]

The Random Forest Classifier achieved a Testing Accuracy of 78.51%, closely followed by GCN at 78.64%. Passive-aggressive and Decision Tree Classifiers performed well, with around 77% Testing Accuracy, while Logistic Regression reached 76.06%. Light GBM had the lowest performance at 70.82%. Traditional machine learning and GCN demonstrated solid results for classifying tweets into emotion categories.

#### Interpretation of dataset 2 [Fig. 7]

The Random Forest Classifier achieved a Testing Accuracy of 88.12%, while GCN excelled with 92.38% .Testing and 92.64% Training Accuracy, showing strong generalization. Passive Aggressive and Decision Tree Classifiers performed well, with Testing Accuracy above 86%, and Logistic Regression followed with 86.32%. However, Light GBM lagged significantly, with the lowest Testing Accuracy at 41.86%. Overall, the Random Forest Classifier of Machine Learning and GNN (GCN) of Deep Learning appear to be the best-performing algorithms for the emotion classification task on the given dataset.

Performance Comparison: Standard vs. Improved GCN

**Table:** 

Model	Graph construction	Feature aggregation	Computational efficiency	Performance on emotion classification
**Standard GCN**	Adjacency matrix (word co-occurrence)	Uniform neighbor aggregation	High efficiency	Limited ability to capture key emotion words
**GAT**	Adjacency matrix	Attention-based aggregation	Computationally expensive	High accuracy but prone to overfitting
**GraphSAGE**	Sampled neighbors	Fixed aggregation (Mean, LSTM, Pooling)	Moderate efficiency	Struggles with fine-grained emotion detection
**Our IGCN**	**PMI-based graph**	**Attention-weighted multi-hop aggregation**	**Improved efficiency over GAT**	**Best balance of accuracy & efficiency**

Our **Improved Graph Convolutional Network (IGCN)** overcomes the limitations of **GCN, GAT, and GraphSAGE** by:

• **Enhancing text representation** using **PMI-based graph construction**.
• **Introducing attention-weighted aggregation** for **better feature importance**.
• **Capturing long-range dependencies** with **multi-hop propagation**.
• **Balancing accuracy and efficiency**, making it **ideal for large-scale social media emotion classification**.

This modification results in **higher classification accuracy** while maintaining **lower computational costs**, making it a **practical choice for real-world applications**.

## Conclusion

This paper focuses on the structural pre-processing of text into graphs and then classifying those graphs into emotions to perform emotion analysis on the text. Two datasets are used in this paper. The results show that graph pre-processing is a viable approach for emotion analysis, which can give access to a robust set of features, including the already available ones. Even with relatively low training data, the problem of overfitting did not occur. GNN shows that graph networks can be a scalable approach to training various natural language models in the future. Graphs can focus on the text's semantic aspects, which is more pertinent to analyzing the sentiment behind the sentence. Our work uses the PMI-based approach for graph pre-processing. Finally, we conclude that we found better testing accuracy, i.e., nearer to 78.64% and 92.38% of both datasets. However, various other techniques can be used in the future to perform graph pre-processing and understand how the models' accuracy varies based on different pre-processing methods. Different GNN models, like recurrent or spatial graph neural networks, can also perform this analysis. In summary, our Improved GCN model offers a scalable, interpretable solution for emotion classification in social media text, outperforming traditional ML models. Future research will explore hybrid GCN-Transformer architectures for even greater accuracy and contextual depth. They can capture words' local and global context, the strength of the relationship between words, and the semantic relation between words. This helps improve the accuracy of sentiment analysis, especially in cases where the relationship between words is not simply binary.

## Limitations

Future research will explore a hybrid model integrating GCNs and Transformers, leveraging the structural understanding of GCNs with the contextual depth of Transformer embeddings (e.g., BERT + Graph Attention Networks). Additionally, real-time implementation on large-scale Twitter streams will be explored for practical deployment.

## Ethics statements

Not applicable.

## CRediT author statement

**BK, SP:** Conceptualization, Methodology, Validity tests, Data curation, Writing- Original draft preparation. **BK, SP, SM, JG:** Visualization, Investigation. **SP, SM:** Supervision. **BK:** Software, Validation. **BK:** Writing- Reviewing and Editing. **SP:** Funding

## Declaration of competing interest

The authors declare that they have no known competing financial interests or personal relationships that could have appeared to influence the work reported in this paper.

## Data Availability

I have shared a Link to my data.

## Appendix Supplementary materials

Application
